# Effects of simvastatin on apolipoprotein M in vivo and in vitro

**DOI:** 10.1186/1476-511X-10-112

**Published:** 2011-07-05

**Authors:** Xiaoying Zhang, Shubing Mao, Guanghua Luo, Jiang Wei, Maria Berggren-Söderlund, Peter Nilsson-Ehle, Ning Xu

**Affiliations:** 1 Comprehensive Laboratory, Third Affiliated Hospital of Suzhou University, Changzhou 213003, China; 2 Division of Clinical Chemistry and Pharmacology, Department of Laboratory Medicine, Lund University, S-221 85 Lund, Sweden

## Abstract

**Objective:**

To investigate effects of lipid lowering drug, simvastatin, on apolipoprotein M expression in the hyperlipidemic mice and in hepatic cell line, HepG2 cells.

**Methods:**

Swiss male mice were randomly divided into the high fat group and control group, and were intragastrically fed with 0.9% saline (control group) or lipid emulsion (high fat group) at the daily dosage of 15 ml/kg body weight, respectively. After 8 weeks feeding, the hyperlipidemic model was successfully induced and these hyperlipidemic mice were then randomly divided into three experimental groups: vehicle control group, high-dose simvastatin-treated group (100 mg/kg body weight), and low-dose simvastatin-treated group (10 mg/kg body weight). Mice were dosed daily for 6 weeks of simvastatin before mice were sacrificed for determining serum lipid profile and apoM protein levels that was determined by using dot blotting analysis. Effects of simvastatin on apoM mRNA expression in the HepG2 cells were determined by real-time RT-PCR.

**Results:**

Comparing to high fat model mice without simvastatin treatment, 100 mg/kg simvastatin could significantly increase serum total cholesterol (*P *< 0.05). Serum apoM levels, in all mice, were significantly lower in the mice at the age of 26 weeks than the mice at 12 weeks old (*P *< 0.05), which indicated that serum apoM levels were significantly correlated to the mice age. It demonstrated also that treatment of simvastatin did not influence serum apoM levels in these mouse model, although serum apoM levels were increased by about 13% in the 10 mg/kg simvastatin group than in the vehicle control group without simvastatin. In HepG2 cell cultures, simvastatin could significantly decrease apoM mRNA levels with dose- and time-dependent manners. At 10 μM simvastatin treatment, apoM mRNA decreased by 52% compared to the controls.

**Conclusion:**

The present study suggested that simvastatin, *in vivo*, had no effect on apoM levels in the hyperlipidemic mouse model. ApoM serum levels in mice were significantly correlated to the animal's age, whereas in cell cultures simvastatin does inhibit apoM expression in the HepG2 cells. The mechanism behind it is not known yet.

## Introduction

Apolipoprotein M(apoM) is one of the latest discovered lipoprotein-associated plasma protein that is mainly synthesized in the liver, and to a smaller amounts, in the kidney [1]. In human plasma, most apoM are found in high-density lipoproteins (HDL) and small proportion present also in the apoB-containing lipoproteins, i.e. chylomicrons, very low-, and low-density lipoproteins (VLDL and LDL) [1,2]. Recent investigation has demonstrated that apoM may participate in the HDL-related biological activities as an important component of HDL particle on the protection of endothelial cells [3]. Wolfrum, et al., [4] reported that apoM is required for preβ-HDL formation and cholesterol efflux to HDL as described for an initial and crucial stage of reverse cholesterol transport, and subsequently protects against atherosclerosis. In addition, the physiological and patho-physiological roles of apoM may also involve in the inflammatory activities and the potential immuno-and inflamm-reactive property of apoM may contribute to the anti-inflammatory function of HDL [5,6]. The statin class of drugs inhibits the enzyme 3-hydroxy-3-methylglutaryl coenzyme A (HMG-CoA) reductase, which is the first committed step of sterol synthesis, lead to a lowering of plasma cholesterol levels. In several large clinical trials, the use of statins reduces coronary events. Simvastatin has been shown to reduce total mortality rates in patients with coronary heart disease [7]. Previous studies have shown that the plasma apoM concentration is positively correlated with leptin levels and negatively correlated with total cholesterol in normal and obese subjects [8]. ApoM gene expression could be directly regulated by transcription factors including transforming growth factor (TGF)-β, hepatic nuclear factor (HNF)-1α, liver receptor homolog (LRH)-1 and forkhead box A2 (Foxa2), all these could regulate hepatic lipid metabolism [9-12]. This may indicate that apoM is involved in lipid and glucose homeostasis. Evidence from different individual laboratories suggests that HMG-CoA reductase inhibitors can down-regulated apoA-IV apoB, apoC-III and apoE, while apoA-I was up-regulated in animal models and cultured hepatocytes [13]. The effect of simvastatin on apoM has not been studied. To examine whether cholesterol-lowering with statin therapy impact on plasma apoM concentration may provide greater insight into the role of apoM in human lipoprotein metabolism. In this study, we investigated if apoM expression is regulated by simvastatin *in vivo *and *in vitro*.

## Materials and methods

### Cells, animals, and reagents

The human hepatoblastoma cell line, HepG2, was obtained from the American Type Culture Collection (ATCC, Manassas, VA). Male Swiss mice were purchased from Shanghai Slac Laboratory Animal Co. (Shanghai, China). Simvastatin was purchased from International Laboratory Co.(Utah, OREM, USA). Cholesterol, Propylthiouracil and Tween-80 were from Sigma Aldrich (St Louis, USA). Deoxycholic acid sodium salt was obtained from Merck (Germany). ABI PRISM 7700 sequence detection system, real-time RT-PCR reagents and control probe GAPDH were purchased from the Applied Biosystems Inc. (Foster City, CA, USA). Rabbit anti-mouse apoM was from Abnova Corporation, Taiwan. Alkaline phosphatase (AP) conjugated Affinipure goat anti-rabbit IgG was from Jackson Immunoresearch Laboratories, Inc., USA. BCIP/NBT Color Development Substrate was from Sino-American Biotechnology Company (Luoyang, China). Quantity One Software V4.6.2 was from Bio-Rad. Cell culture flasks (25 cm^2^) and 6-well cell culture clusters were from Costar, USA.

### Animal experiments

Fifty male Swiss mice weighing 30-32 g, at 12-weeks old, were subjected in the study. Mice were acclimatized one week prior to the experiment and were housed in standard cages at 22°C and 40-60% relative humidity with a 12-hrs light/dark cycle, maintained on standard chow and water ad libitum. The mice were randomly assigned to and housed in ten different cages containing sawdust bedding. For inducing hyperlipidemia, mice were fed on a lipid emulsion that contains 20% (g/100 g) fat of lard, 10% cholesterol, 2% deoxycholic acid sodium salt, 1% propylthiouracil, 10% Tween-80 and double-distilled water. Mice were randomly divided into two groups, i.e., high fat group (fed lipid emulsion) and control group (fed 0.9% saline) at the daily dosage of 15 ml/kg body weight, respectively. After 8 weeks feeding, thirty-nine hyperlipidemic mice successfully induced, which were randomly divided into three experimental groups. And then animals were dosed daily via oral gavage with 10 or 100 mg/kg simvastatin in 0.5% methylcellulose or administered methylcellulose alone (vehicle control) starting at 20 weeks of age. After 6 weeks, mice were anesthetized and sacrificed. Blood samples were taken through tail vein and the serum was centrifuged and stored at -80°C. The housing care of the animals and all the procedures used in these studies were performed in accordance with the guidelines and regulations of the University of Suzhou Institutional Animal Care and Use Committee.

### Cell cultures

HepG2 cells were grown in RPMI 1640 with 10% fetal calf serum (FCS) in the presence of benzylpenicillin (100 U/ml) and streptomycin (100 μg/ml) under standard culture conditions (5% CO_2_, 37°C). Cells were seeded in 25-cm^2 ^cell culture flasks or in 6-well cell culture clusters, and grown to 50-70% confluence.

Prior to experiments, cells were washed twice with phosphate buffered saline (PBS) and once with serum-free RPMI 1640 without antibiotics. Then the experimental medium, containing RPMI 1640 with 0.5% human serum albumin (HSA) and different concentrations of simvastatin (0 μM, 3 μM, 5 μM,10 μM, and 30 μM) were added. Cells were incubated at 37°C with different time intervals.

### Isolation of total RNA and real-time RT-PCR

Total RNA of HepG2 cells was isolated by the guanidinium thiocyanate method [14]. Primer Express software (Applied Biosystems) was used to design human apoM primers and probes based on the TaqMan assay. In order to avoid amplifying the DNA template, the apoM specific primers that are spanned an 81-bp intron was designed to amplify a 66-bp product. The primers were 5'-tgccccggaaatggatcta and 5'-cagggcggccttcagtt, and the probe was 5'-FAM-cacctgactgaagggagcacagatctca-TAMRA. Relative standard curves for apoM and GAPDH were performed to compensate for the efficiency of PCR. A serial dilution of human apoM cDNA was used to generate a standard curve by plotting the cycle threshold versus the log of input cDNA. The apoM and GAPDH standards were linear with the input of cDNA. Quantification of apoM mRNA levels is relative to GAPDH mRNA levels, and was performed on an ABI PRISM 7700 Sequence Detector. The real-time RT-PCR was performed in two steps in a 25 μl reaction mixture containing 1 μl TaqMan Universal PCR Master Mix, 22.5 pmol of both forward and reverse primers, 5 pmol probes and 50 ng of the total RNA templates. Thermal cycling conditions included the following steps: 25°C 10 min, 48°C 30 min, and 95°C 5 min to do reverse transcription, and then the reaction mixture was preheated for 2 min at 50°C and for 10 min at 95°C to activate Taq polymerase. After that, a 40-cycle two-step PCR was performed consisting of 15s at 95°C and 1 min at 60°C. All experiments were performed at least three times in triplicate.

### Determination of serum apoM

Serum apoM levels were semi-quantitatively examined by a dot-blot analysis with a specific rabbit anti-mouse apoM antibody. Two-microliter serum samples were applied to the Hybond-C membrane in triplicate. All samples were applied to the same membrane. The membrane was quenched in Tris-HCl buffer in presence of 4% Tween and 3% BSA for 3 hrs, and sequentially incubated with primary antibody (1:1000 dilution in Tris-HCl buffer) overnight at 4°C. After washing by Tris-HCl buffer three times membrane was then incubated with AP conjugated secondary antibody for 2 hrs at room temperature. The development of AP activity was performed with a commercial visualization system according to the manufacturer's instructions. The relative amount of apoM were analyzed with Quantity One Software, and presented as volume (intensity*mm^2^).

### Statistical analyses

Statistical analysis was performed with Graphpad Prism 5.0 software (GraphPad Software Inc.). Results are expressed as means ± SEM. Multiple comparisons were performed with one-way ANOVA/Turkey, and comparisons between after and before simvastatin treatment of each group were statistically evaluated by the paired *t*-test. Significance was established at a *P *value less than 0.05.

## Results

### Serum lipid profile in mice after a high fat diet and effects of simvastatin on serum levels of lipid and apoM

**Figure 1:**
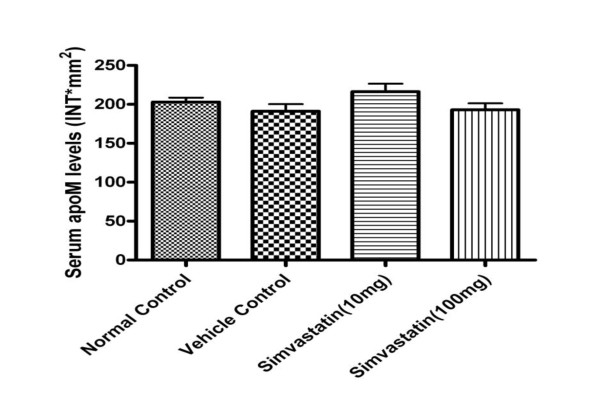
**Serum apoM levels in mice treated with simvastatin for 6 weeks**. ApoM concentrations were determined by dot-blotting analyses as described in the materials and methods. Data are expressed as the intensity*mm^2 ^that was analyzed by the software of Quantity One. Data are means ± SEM.

**Figure 2:**
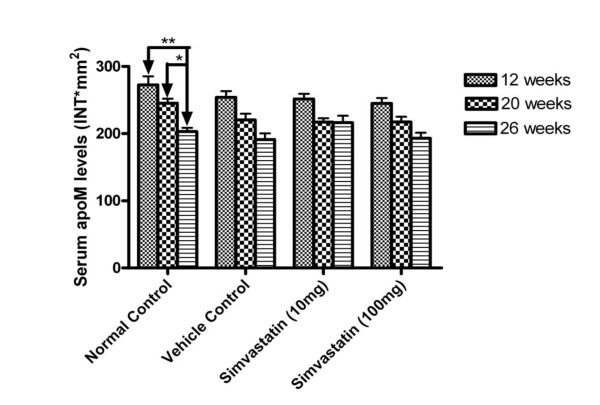
**Serum apoM levels in mice at different time intervals**. ApoM concentrations were determined by dot-blotting analyses as described in the materials and methods. Data are expressed as the intensity*mm^2 ^that was analyzed by the software of Quantity One. Data are means ± SEM. *P < 0.05 vs. 20-week normal control, **P < 0.01 vs. 12-week normal control.

As shown in table 1, the serum profile of LDL-cholesterol, total cholesterol were significantly increased in the mice under oral gavaged for 8 weeks compared to the control group by 154% and 85%, respectively. Serum triglycerides were didn't change after 12 weeks high fat feeding, whereas after 20 weeks feeding, serum triglycerides were even slightly decreased in these mice compared to controls. In the hyperlipidemic mice after treated with simvastatin, either in the low dose (10 mg/kg body weight) or high dose (100 mg/kg body weight), unexpectedly, serum total cholesterol levels were significantly increased compared to the vehicle control mice and normal control mice (Table 2). The same phenomenon was seen in LDL-cholesterol levels too. As shown in Figure 1, there were no statistical significant differences on serum apoM levels in these mice at 26 weeks, although serum apoM levels were slightly increased (about 13%) in the 10 mg/kg simvastatin-treated mice compared to the vehicle control mice (Figure 1). As shown in Figure 2, serum apoM levels, in all mice, were dramatically lower in the mice at the age of 26 weeks than in the mice at 12 weeks old (*P *< 0.05). At the 26 th week, serum apoM levels were decreased by 26% (*P *< 0.01) and 17% (*P *< 0.05), respectively, compared to 12 or 20 weeks of normal control mice. It is also demonstrated that neither low dose (10 mg/kg body weight) nor high dose (100 mg/kg body weight) influence serum apoM levels.

**Table 1:** Serum lipid profile in mice fed a high fat diet

Lipids (mmol/L)		Control diet			High fat diet	
	
	12 weeks	20 weeks	*P*	12 weeks	20 weeks	*P*
**TC**	2.44 ± 0.22	2.64 ± 0.20	*P *> 0.05	2.97 ± 0.09	4.89 ± 0.18	*P *< 0.0001
**LDL-C**	0.40 ± 0.03	0.40 ± 0.02	*P *> 0.05	0.47 ± 0.01	0.99 ± 0.05	*P *< 0.0001
**HDL-C** **TG**	1.53 ± 0.10 1.53 ± 0.10	1.61 ± 0.12 1.67 ± 0.15	*P *> 0.05 *P *> 0.05	1.73 ± 0.06 1.57 ± 0.07	1.69 ± 0.06 0.98 ± 0.04	*P *> 0.05 *P *< 0.0001

Data are shown as means ± SEM. TC (total cholesterol), HDL-C (high density lipoprotein cholesterol), LDL-C (low density lipoprotein cholesterol), TG (triglyceride).

**Table 2:** Effects of simvastatin on serum lipid levels (mmol/L)

Groups	N	TC	TG	HDL-C	LDL-C
**Normal control**	6	2.39 ± 0.17	2.30 ± 0.26	0.27 ± 0.03	0.26 ± 0.02
**Vehicle control**	9	3.59 ± 0.22^**a**^	1.58 ± 0.11^**b**^	1.17 ± 0.10^**b**^	0.73 ± 0.06^**b**^
**Simvastatin (10 mg/kg)**	10	3.84 ± 0.18	1.19 ± 0.08	1.34 ± 0.12	0.76 ± 0.04
**Simvastatin (100 mg/kg)**	14	4.49 ± 0.25*	1.33 ± 0.09	1.59 ± 0.17	0.93 ± 0.09

Data are shown as means ± SEM. TC (total cholesterol), HDL-C (high density lipoprotein cholesterol), LDL-C (low density lipoprotein cholesterol), TG (triglyceride). *P < 0.05 vs. vehicle control, ^a^*P *< 0.05 vs. normal control, ^b^*P *< 0.01 vs. normal control.

### Effects of simvastatin on apoM mRNA levels in HepG2 cell cultures

**Figure 3:**
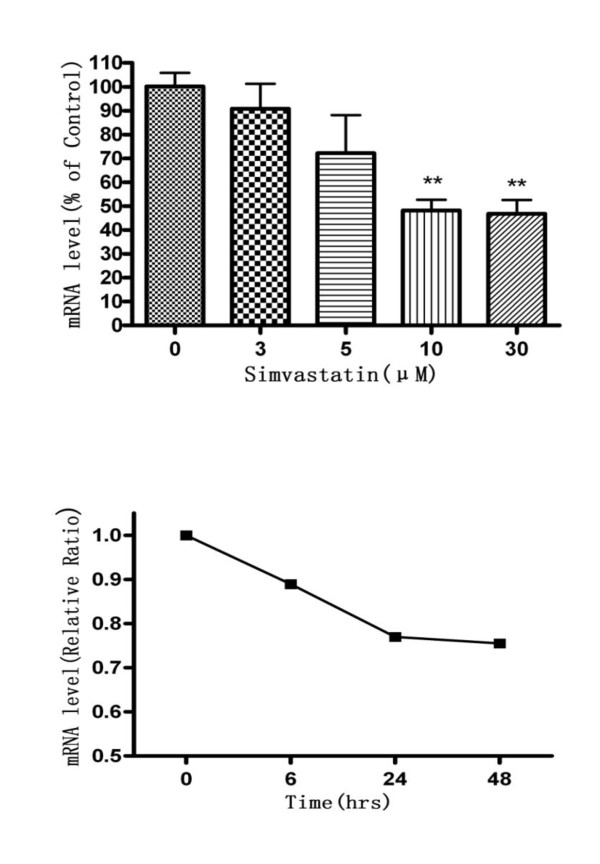
**Effects of simvastatin on apoM mRNA levels in HepG2 cells**. HepG2 cells were cultured with different concentrations of simvastatin for 24 hrs (upper panel) or cells were cultured with 30 μM simvastatin up to 48 hrs (lower panel). ApoM mRNA levels were determined by real-time RT-PCR. Each experimental group contains six replicates and real-time RT-PCR was run in triplicates. The control group is represented as 100%. Data are means ± SEM. **P < 0.01 vs. control.

As shown in Figure 3, simvastatin could significantly inhibited apoM expression in the HepG2 cells, with dose- and time-dependent manners. Simvastatin at 10 μM decreased apoM mRNA in HepG2 cells by about 52% compared to the controls (*P *< 0.01) (Figure 3).

## Discussion

ApoM is one of the latest discovered human apolipoprotein predominantly present in HDL in human plasma, and its physiological and patho-physiological roles remain to be clarified [1]. Experiments in transgenic mice suggested apoM may have anti-atherogenic properties and the possible mechanisms include increased formation of pre-β HDL, enhanced cholesterol mobilization from foam cells, and increased antioxidant properties [2,4]. Statins, inhibitors of 3-hydroxy-3-methylglutaryl coenzyme A (HMG-CoA) reductase, have revolutionized the treatment of hypercholesterolemia. They are the most efficient agents for reducing plasma cholesterol, being also appreciated for their good tolerance. Angiographic studies have demonstrated that these compounds reduce the progression and may induce the regression of atherosclerotic lesion in cardiovascular vessels, which could lead to a significant decreases of cardiovascular morbidity and mortality [15]. Statins have been shown to inhibit hepatic production of apoA-IV apoB, apoCIII and apoE-containing lipoproteins both *in vitro *and *in vivo *[13,16].

In the present study, we demonstrated that simvastatin, *in vivo*, had no effect on apoM levels in the hyperlipidemic mouse model or in normal mice. Interestingly apoM serum levels in mice were significantly correlated to the animal's age. In wild-type mouse plasma, cholesterol was predominantly found in HDL and apoM was also recovered in the same fractions as HDL [17]. This may partly explain the reason why normal control mice had a pronounced decrease in HDL cholesterol [18]. The detailed mechanism needs further investigation. However, in HepG2 cell cultures, simvastatin does inhibit apoM expression. We don't have the reasonable explanation on such difference between the animal models and cell cultures. In addition, in the present study, we demonstrated that serum triglyceride levels were significantly decreased in the high fat diet group mice compared to normal control mice at 20 weeks, which may be possible that the high fat diets contain some cholates. As a bile salt, it is a ligand for the nuclear hormone receptor FXR, whose activity regulates the expression of multiple genes involved in lipoprotein metabolism. Among these genes are apoCII and apoCIII, whose alterations of expression could account for the fact that the presence of cholate in the diet is associated with a lower plasma triglyceride than is the case for similar diets without cholate [19]. It is interesting to note that simvastatin could elevate serum cholesterol in Swiss mice when dosed at 100 mg/kg body weight/daily for 6 weeks in this study, which is consistent with previous observations by Wang et al [20], who reported that simvastatin could increase serum cholesterol in mice. The mechanism responsible for this unexpected increase of serum cholesterol is unknown yet. One possible explanation may be related to the discrepancies of various species. It may also be possible that higher continuous dose of simvastatin had stimulatory effects on the expression and activity of 3-hydroxy-3-methylglutaryl coenzyme A reductase in the liver [21], which lead to an increased cholesterol levels in these mice. The detailed mechanism needs further investigation.

## Competing interests

The authors declare that they have no competing interests.

## Authors' contributions

XZ, SM, GL and JW carried out experiments, data collection, performed the statistical analysis and drafted the manuscript. MBS, PNE and NX participated in the design of the experiments and helped to draft the manuscript. All authors read and approved the final manuscript.

## References

[B1] Xu N Dahlback B A novel human apolipoprotein (apoM) J Biol Chem 1999 274 44 31286 31290 10.1074/jbc.274.44.31286 10531326

[B2] Christoffersen C Nielsen LB Axler O Andersson A Johnsen AH Dahlback B Isolation and characterization of human apolipoprotein M-containing lipoproteins J Lipid Res 2006 47 8 1833 1843 10.1194/jlr.M600055-JLR200 16682745

[B3] Christoffersen C Obinata H Kumaraswamy SB Galvani S Ahnstrom J Sevvana M Egerer-Sieber C Muller YA Hla T Nielsen LB Dahlbäck B Endothelium-protective sphingosine-1-phosphate provided by HDL-associated apolipoprotein M Proc Natl Acad Sci USA 2011 10.1073/pnas.1103187108 PMC3111292 21606363

[B4] Wolfrum C Poy MN Stoffel M Apolipoprotein M is required for prebeta-HDL formation and cholesterol efflux to HDL and protects against atherosclerosis Nat Med 2005 11 4 418 422 10.1038/nm1211 15793583

[B5] Christoffersen C Jauhiainen M Moser M Porse B Ehnholm C Boesl M Dahlback B Nielsen LB Effect of apolipoprotein M on high density lipoprotein metabolism and atherosclerosis in low density lipoprotein receptor knock-out mice J Biol Chem 2008 283 4 1839 1847 18006500 10.1074/jbc.M704576200

[B6] Venteclef N Haroniti A Tousaint JJ Talianidis I Delerive P Regulation of anti-atherogenic apolipoprotein M gene expression by the orphan nuclear receptor LRH-1 J Biol Chem 2008 283 7 3694 3701 17977826 10.1074/jbc.M706382200

[B7] Randomised trial of cholesterol lowering in 4444 patients with coronary heart disease: the Scandinavian Simvastatin Survival Study (4S) Lancet 1994 344 8934 1383 1389 7968073

[B8] Xu N Nilsson-Ehle P Ahren B Correlation of apolipoprotein M with leptin and cholesterol in normal and obese subjects J Nutr Biochem 2004 15 10 579 582 10.1016/j.jnutbio.2004.03.001 15542348

[B9] Richter S Shih DQ Pearson ER Wolfrum C Fajans SS Hattersley AT Stoffel M Regulation of apolipoprotein M gene expression by MODY3 gene hepatocyte nuclear factor-1alpha: haploinsufficiency is associated with reduced serum apolipoprotein M levels Diabetes 2003 52 12 2989 2995 10.2337/diabetes.52.12.2989 14633861

[B10] Xu N Hurtig M Zhang XY Ye Q Nilsson-Ehle P Transforming growth factor-beta down-regulates apolipoprotein M in HepG2 cells Biochim Biophys Acta 2004 1683 1-3 33 37 15238217 10.1016/j.bbalip.2004.04.001

[B11] Venteclef N Smith JC Goodwin B Delerive P Liver receptor homolog 1 is a negative regulator of the hepatic acute-phase response Mol Cell Biol 2006 26 18 6799 6807 10.1128/MCB.00579-06 16943422 PMC1592867

[B12] Wolfrum C Howell JJ Ndungo E Stoffel M Foxa2 activity increases plasma high density lipoprotein levels by regulating apolipoprotein M J Biol Chem 2008 283 24 16940 16949 10.1074/jbc.M801930200 18381283

[B13] Mitchell A Fidge N Griffiths P The effect of the HMG-CoA reductase inhibitor simvastatin and of cholestyramine on hepatic apolipoprotein mRNA levels in the rat Biochim Biophys Acta 1993 1167 1 9 14 8461338 10.1016/0005-2760(93)90210-z

[B14] Chomczynski P Sacchi N Single-step method of RNA isolation by acid guanidinium thiocyanate-phenol-chloroform extraction Anal Biochem 1987 162 1 156 159 2440339 10.1006/abio.1987.9999

[B15] Vaughan CJ Gotto AM Jr Basson CT The evolving role of statins in the management of atherosclerosis J Am Coll Cardiol 2000 35 1 1 10 10.1016/S0735-1097(99)00525-2 10636252

[B16] Bonn V Cheung RC Chen B Taghibiglou C Van Iderstine SC Adeli K Simvastatin, an HMG-CoA reductase inhibitor, induces the synthesis and secretion of apolipoprotein AI in HepG2 cells and primary hamster hepatocytes Atherosclerosis 2002 163 1 59 68 10.1016/S0021-9150(01)00754-7 12048122

[B17] Faber K Axler O Dahlback B Nielsen LB Characterization of apoM in normal and genetically modified mice J Lipid Res 2004 45 7 1272 1278 10.1194/jlr.M300451-JLR200 15102887

[B18] Camus MC Chapman MJ Forgez P Laplaud PM Distribution and characterization of the serum lipoproteins and apoproteins in the mouse, Mus musculus J Lipid Res 1983 24 9 1210 1228 6631247

[B19] Getz GS Reardon CA Diet and murine atherosclerosis Arterioscler Thromb Vasc Biol 2006 26 2 242 249 16373607 10.1161/01.ATV.0000201071.49029.17

[B20] Wang YX Martin-McNulty B Huw LY da Cunha V Post J Hinchman J Vergona R Sullivan ME Dole W Kauser K Anti-atherosclerotic effect of simvastatin depends on the presence of apolipoprotein E Atherosclerosis 2002 162 1 23 31 10.1016/S0021-9150(01)00678-5 11947894

[B21] Bea F Blessing E Bennett B Levitz M Wallace EP Rosenfeld ME Simvastatin promotes atherosclerotic plaque stability in apoE-deficient mice independently of lipid lowering Arterioscler Thromb Vasc Biol 2002 22 11 1832 1837 10.1161/01.ATV.0000036081.01231.16 12426212

